# Gastritis Cystica Profunda With High‐Grade Dysplasia Mimicking Gastrointestinal Stromal Tumor: A Histopathological Pitfall in the Assessment of Submucosal Invasion

**DOI:** 10.1155/crgm/7741186

**Published:** 2026-10-20

**Authors:** Vy Thi-Thuy Duong, Anh The Phan, Tho Ngoc-Quynh Le, Huyen Thi Thanh Le, Giang Huong Tran

**Affiliations:** ^1^ Department of Pathology University Medical Center Ho Chi Minh City Ho Chi Minh City 700000 Vietnam bvdaihoc.com.vn; ^2^ Department of Histology Embryology, and Pathology School of Medicine University of Medicine and Pharmacy at Ho Chi Minh City Ho Chi Minh City 700000 Vietnam hcmut.edu.vn; ^3^ Independent Researcher Ho Chi Minh City 700000 Vietnam

**Keywords:** gastric high-grade dysplasia, gastritis cystica profunda, gastrointestinal stromal tumor, muscularis mucosae, staging, submucosal tumor

## Abstract

**Background:**

Gastritis cystica profunda (GCP) is an uncommon gastric lesion characterized by displacement of cystically dilated glands into the submucosa. It may present as a submucosal mass mimicking gastrointestinal stromal tumor (GIST). The coexistence of GCP with high‐grade dysplasia is extremely uncommon and may pose a significant diagnostic challenge by simulating invasive carcinoma.

**Case Presentation:**

We report a case of a 54‐year‐old patient whose endoscopic and radiographic features suggested a GIST in the gastric cardia. Surgical resection was performed. Histopathological examination revealed cystically dilated glands extending into the submucosa, lined by high‐grade dysplastic epithelium. Importantly, the muscularis mucosae was focally disrupted, and the submucosal component comprised both dysplastic and nondysplastic glands displaced together within smooth muscle bundles derived from the muscularis mucosae and accompanied by lamina propria‐type stroma, supporting mucosal prolapse rather than true invasion. A final diagnosis of high‐grade dysplasia arising in GCP was established.

**Conclusion:**

This case highlights an important diagnostic pitfall in distinguishing pseudoinvasion from true invasive carcinoma in submucosal gastric lesions. Recognition of this entity and careful assessment of the muscularis mucosae together with the character of the displaced glands are essential to avoid overstaging and unnecessary aggressive management.

## 1. Introduction

Gastritis cystica polyposa or profunda (GCP) is characterized by the displacement of glands into the muscularis mucosae or deeper layers of the gastric wall [1, 2]. Clinically, it may present as a submucosal mass and can closely mimic gastrointestinal stromal tumor (GIST) or, less commonly, gastric carcinoma on endoscopic and radiologic evaluation.

The pathogenesis of GCP remains incompletely understood. It has been associated with prior gastric surgery, as well as chronic gastritis [3]. Several cases have been reported in patients without a history of surgery or infection [3, 4], indicating the contribution of additional, yet unidentified, factors to the development of glandular displacement.

Although GCP is generally considered a benign condition, its association with epithelial dysplasia or adenocarcinoma has been increasingly recognized. In such cases, displacement of dysplastic glands into the submucosa may simulate invasive carcinoma, resulting in a significant diagnostic pitfall and potential overtreatment.

Herein, we report a case of high‐grade dysplasia arising in GCP presenting as a submucosal gastric mass mimicking GIST and highlight the key clinicopathological features that help distinguish pseudoinvasion from true invasion.

## 2. Case Presentation

A 54‐year‐old man presented with a 6‐month history of dull epigastric pain associated with bloating and regurgitation. He denied weight loss or functional limitation. His medical history was notable for prior appendectomy and abdominal hernia repair.

Upper gastrointestinal endoscopy revealed a submucosal lesion with a smooth, intact overlying mucosal surface in the gastric cardia (Figure 1). A Helicobacter pylori (CLO) test was positive. Contrast‐enhanced abdominal computed tomography demonstrated a well‐circumscribed, heterogeneous lesion measuring 22 mm, without lymphadenopathy or adjacent tissue infiltration (Figure 1). On unenhanced images, the lesion was hypoattenuating relative to the adjacent gastric wall, with a mean attenuation of 37 HU (SD 16.5; range 16–73 HU) within a region of interest manually placed by a single radiologist over the solid, enhancing portion of the lesion, deliberately excluding the nonenhancing cystic component and the lesion margin to minimize partial‐volume averaging. It showed progressive and strong enhancement, with the mean attenuation rising to 70 HU (SD 14.8; range 39–91 HU) in the arterial phase and to 108 HU (SD 11.3; range 87–130 HU) in the venous phase, a hypervascular pattern consistent with a GIST. A well‐defined area within the lesion showed no enhancement in any phase, suggesting a cystic component. Endoscopic ultrasonography was not performed: The endoscopic and computed tomographic findings were already considered sufficient to establish an indication for resection of a suspected GIST, and the multidisciplinary tumor board therefore recommended proceeding directly to surgery. Endoscopic biopsy showed chronic active gastritis. Based on these findings, a GIST was suspected, and the patient underwent proximal partial gastrectomy following multidisciplinary tumor board discussion. Gross examination revealed a soft, whitish lesion containing multiple mucus‐filled cysts of varying sizes (Figure 1).

**FIGURE 1:**
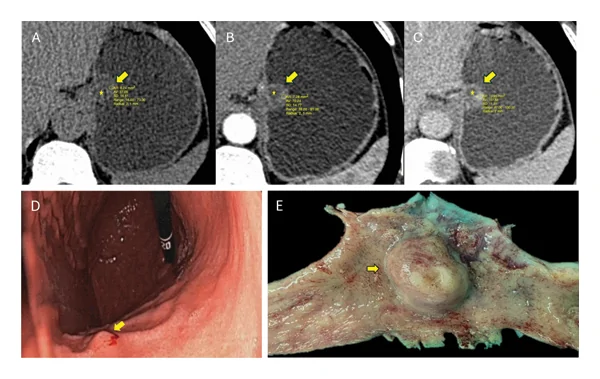
Clinical and imaging features of a gastric submucosal lesion mimicking gastrointestinal stromal tumor (GIST). (A) Unenhanced abdominal CT image showing the lesion in the gastric cardia. (B, C) Contrast‐enhanced abdominal CT images obtained during the arterial (B) and venous (C) phases demonstrate a well‐circumscribed submucosal lesion along the lesser curvature of the gastric cardia (arrows), bulging into the gastric lumen. The lesion shows progressive and strong enhancement from the arterial to the venous phase, consistent with a hypervascular lesion. The circle indicates the region of interest placed in the solid enhancing component (mean attenuation 37, 70, and 108 HU on unenhanced, arterial, and venous phase images, respectively). A hypoattenuating area within the lesion shows no contrast enhancement (asterisk), suggesting a cystic component. (D) Endoscopic examination showing an approximately 20‐mm submucosal lesion in the gastric cardia with a smooth overlying mucosal surface. (E) Gross examination confirming a well‐circumscribed submucosal mass (arrow).

Histopathological examination revealed cystically dilated glands with high‐grade dysplasia extending into the submucosa. However, at low magnification, the glandular proliferation was largely circumscribed by bundles of smooth muscle that could be traced directly into the muscularis mucosae of the adjacent nonlesional mucosa. The muscularis mucosae itself was focally discontinuous, and dilated glands were seen herniating into the submucosa through these defects (Figure 2A). Recognition that the displaced glands were of prolapse type rather than invasive rested on a constellation of findings (Figure 2B). First, the submucosal component comprised both dysplastic glands and nondysplastic, cystically dilated glands lined by bland columnar epithelium, displaced together within the same smooth muscle‐bounded compartment; invasive carcinoma does not carry non‐neoplastic glands with it into the stroma. Second, the displaced glands were accompanied by lamina propria‐type stroma and evoked no desmoplastic reaction. Third, they retained rounded, smoothly contoured profiles and a lobular arrangement, with no angulated glands, no single infiltrating cells, and no tumor budding identified anywhere in the lesion. Fourth, the dysplastic epithelium lining the submucosal glands was cytologically identical to, and in continuity with, the overlying surface epithelium. Taken together, these features indicated mucosal prolapse (pseudoinvasion) rather than true stromal invasion. Accordingly, the lesion was diagnosed as high‐grade dysplasia arising in GCP rather than invasive adenocarcinoma. The dysplastic glands were continuous with the surface epithelium, with no lymphovascular or perineural invasion identified. Immunohistochemistry demonstrated a high Ki‐67 proliferation index (Figure 3B) and a p53‐null pattern (Figure 3C). Epstein–Barr virus in situ hybridization was negative (Figure 3D).

**Figure 2:**
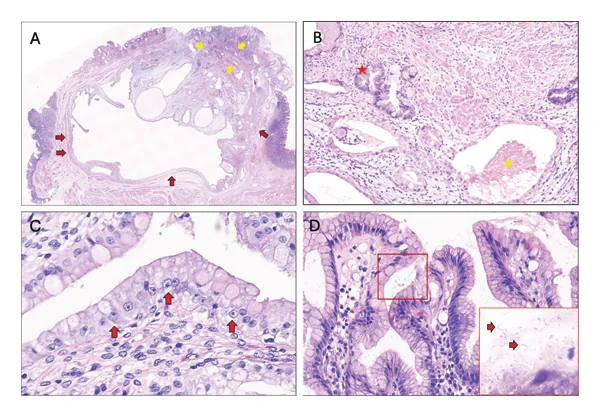
Histopathological features of gastritis cystica profunda‐associated high‐grade dysplasia. (A) Low‐power view showing prolapse of gastric mucosa into the submucosa. The displaced glands are enveloped by bundles of smooth muscle extending downward from the muscularis mucosae (red arrows). The muscularis mucosae is focally discontinuous, with glands herniating into the submucosa through these defects (yellow arrows) (50*x*). (B) Higher magnification of the submucosal component shown in (A). Dysplastic glands lined by hyperchromatic, pseudostratified epithelium (red star) lie immediately adjacent to nondysplastic, cystically dilated glands lined by bland columnar epithelium and containing inspissated mucin (yellow star). Both are displaced together within the same smooth muscle‐bounded compartment and are accompanied by lamina propria‐type stroma, without a desmoplastic reaction (100*x*). (C) High‐power view of the dysplastic epithelium, composed of cells with enlarged oval nuclei, vesicular chromatin, and prominent nucleoli (arrows) (400*x*). (D) *Helicobacter pylori*–associated chronic gastritis in the mucosa adjacent to the lesion; the boxed area marks the surface mucus shown at higher magnification in the inset (200*x*). Inset: *Helicobacter pylori* organisms within the surface mucus (arrows, 1000*x*).

**FIGURE 3:**
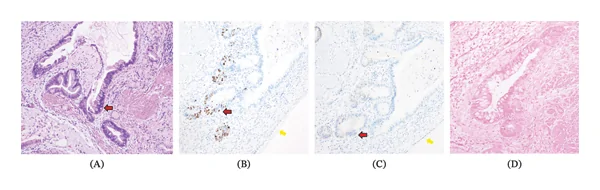
Immunohistochemical and *in situ* hybridization features of high‐grade dysplasia arising in gastritis cystica profunda. (A) Dysplastic glands with atypical epithelial cells embedded within submucosal smooth muscle bundles (arrow), lying adjacent to nondysplastic displaced glands (H&E, 100*x*). (B) Ki‐67 immunostaining showing an increased proliferation index in the dysplastic glands (red arrow) compared with the adjacent nondysplastic glands (yellow arrow) (100*x*). (C) p53 immunostaining demonstrating a null pattern, with complete absence of nuclear staining in the dysplastic glands (red arrow), in contrast to the wild‐type pattern retained in the adjacent nondysplastic glands (yellow arrow) (100*x*). (D) Epstein–Barr virus–encoded small RNA in situ hybridization showing no nuclear signal in the dysplastic glands, indicating a negative result (Nuclear Fast Red Counterstain, 100*x*).

Based on the overall clinicopathological findings, a final diagnosis of gastric high‐grade dysplasia arising in GCP was established. No additional treatment was administered. At 13‐month follow‐up, the patient remained free of recurrence or lymphadenopathy.

## 3. Discussion

Malignant transformation of GCP has not been well characterized. Reported cases of GCP‐associated high‐grade dysplasia and adenocarcinoma are rare and predominantly affect older male patients, with a median age of approximately 60 years [5, 6] (Table 1). Lesions most commonly arise in the proximal stomach, particularly the fundus and corpus, and frequently present as submucosal masses that mimic other neoplasms. These findings are consistent with our case, in which the lesion presented as a submucosal mass clinically suspected to be a GIST.

**TABLE 1:** Clinicopathological features of GCP from the PubMed electronic database from January 1947 to August 2025.

No.	Histologic analysis	Authors	Reported year	Age	Sex	Location of mass	Clinical diagnosis	Size (cm)	Imaging findings	Treatment	History	Follow‐up time	*H. pylori*
1	Adenocarcinoma	Mitomi et al. [7]	1998	51	M	Corpus	Gastric cancer	4.8	Exophytic mass	Surgical resection	No surgery	120 months, NED	Negative
2	Mixed adenocarcinoma (tub, muc, sig)	Tsuji et al. [8]	2008	61	M	Corpus	Gastric cancer	ND	Endoscopy: 0‐IIc	Surgical resection	Peptic ulcer (no surgery)	36 months, NED	Positive
3	Adenocarcinoma	Moon et al. [9]	2010	77	M	Antrum	Gastric cancer	0.6	Endoscopy: Depressed nodular lesions	EMR/ESD	Previous ESD 4 months earlier	24 months, NED	Positive
4	Carcinoma with lymphoid stroma	Kim et al. [10]	2012	50	M	Corpus	Early gastric cancer	3.0	Early gastric cancer	Surgical resection	No	18 months, NED	Positive
5	Adenocarcinoma	Deery et al. [11]	2012	70	F	Antrum	Gastric cancer	1.5	A broad‐based polypoid lesion with central ulceration	Surgical resection	No surgery	6 months, NED	Negative
6	Adenocarcinoma	Matsumoto et al. [12]	2012	51	F	Antrum	Submucosal tumor	7.0	A multicystic mass in the submucosal layer	Surgical resection	No surgery	ND	ND
7	Adenocarcinoma	Lee et al. [13]	2013	77	M	Corpus	Early gastric cancer	1.5	Submucosal tumor	EMR/ESD	ESD	NA	NA
8	Adenocarcinoma	Kuwahara et al. [14]	2013	63	M	Corpus	Advanced gastric cancer	ND	Ulcerating lesion	Surgical resection	No	ND	ND
9	Adenocarcinoma	Ogasawara et al. [15]	2014	55	M	Corpus	Submucosal tumor	1.2	Submucosal tumor	EMR/ESD	No surgery	39 months, NED	Negative
10	Mixed (tub + sig) adenocarcinoma	Namikawa et al. [16]	2017	83	F	Gastric side of the anastomosis of the gastrojejunostomy	Gastric cancer	9.5	An irregular nodular lesion	Surgical resection	Gastrojejunostomy with Braun jejunojejunostomy for benign chronic peptic ulcer	46 months, NED	ND
11	Adenocarcinoma	Huang et al. [17]	2018	61	M	Cardia	Possibility of gastric carcinoma	0.9	NBI: mucosal irregular depression with marginal nodular elevation in the posterior wall of the gastric body and fundus junction	EMR/ESD	Surgery for duodenal ulcer and perforation 1 year earlier	5 months, NED	Positive
12	Adenocarcinoma	Wahi et al. [18]	2020	45	M	Corpus	GIST	5.2	An exophytic mass	Surgical resection	No	ND	Negative
13	High‐grade dysplasia	Wang et al. [19]	2022	64	F	Fundus	Polyp	0.8	Polyp	EMR/ESD	No	39 months, NED	ND
14	Adenocarcinoma	Wang et al. [19]	2022	34	M	Corpus	Submucosal tumor	1.1	Submucosal tumor	EMR/ESD	No	8 months, NED	ND
15	Adenocarcinoma	Eriksson et al. [20]	2024	45	F	Corpus	Advanced gastric cancer	ND	Submucosal tumor	Surgical resection	No	ND	Positive
16	Adenocarcinoma	Huang and Liu [21]	2025	77	M	Corpus	Gastric cancer	2.3	Ulcers	EMR/ESD	No surgery	10 months, NED	Negative
17	High‐grade dysplasia	Our case	2025	54	M	Cardia	Submucosal tumor	2.2	Submucosal tumor	Surgical resection	No gastric surgery	13 months, NED	Yes

*Note:* M: male; F: female.

Abbreviations: EMR, endoscopic mucosal resection; ESD, endoscopic submucosal dissection; NBI, narrow band imaging; ND, no data; NED, no evidence of disease.

Including the present patient, 17 cases of GCP‐associated dysplasia or carcinoma have now been reported (Table 1). Fifteen were invasive adenocarcinoma, including one carcinoma with lymphoid stroma and two mixed‐type tumors, whereas high‐grade dysplasia without invasion has been documented only once before [19]; our case is therefore the second. Lesions ranged from 0.6 to 9.5 cm and arose predominantly in the corpus, with three in the antrum, two at the cardia and one at a gastrojejunal anastomosis. Only three patients had undergone previous gastric or duodenal surgery, so the majority, like ours, developed GCP in an unoperated stomach, most often in association with *Helicobacter pylori* infection or peptic ulcer disease. It should be emphasized that GCP is not merely an incidental finding: It arises characteristically at gastroenterostomy sites after Billroth II reconstruction and is recognized as a substrate for remnant gastric carcinoma, and the accumulated reports summarized here indicate that it should be regarded as a lesion with genuine, if low, neoplastic potential rather than a purely reactive process. Notably, most previous reports did not state whether the dysplastic or carcinomatous glands were confined to the cystically dilated submucosal glands or were also present in the overlying mucosa, nor did they describe the relationship between the neoplastic glands and the muscularis mucosae. The present case is unusual in documenting both: The dysplastic epithelium lined the displaced submucosal glands and was continuous with the surface epithelium, and the displaced glands, dysplastic and nondysplastic alike, remained within smooth muscle derived from the muscularis mucosae despite its focal discontinuity. Regarding treatment, endoscopic resection was used in 7 of the 17 cases and surgical resection in 10; endoscopic resection was generally chosen for smaller lesions (0.6–2.3 cm) confined to the mucosa and submucosa, whereas gastrectomy was reserved for larger tumors, deeply invasive carcinoma, or anastomotic lesions. On this basis, endoscopic submucosal dissection appears appropriate for GCP‐associated dysplasia or intramucosal carcinoma when the lesion is small, superficial, and adequately characterized preoperatively, and surgical resection for larger or invasive lesions.

From a clinical perspective, GCP‐associated neoplastic lesions often present as submucosal masses with nonspecific endoscopic and radiologic features. The size of GCP‐associated adenocarcinoma varies widely, ranging from 0.6 to 9.5 cm [5, 6]. Endoscopically, these lesions may appear as early or advanced gastric cancer, or present as submucosal masses. Although endoscopic ultrasonography may demonstrate a multicystic pattern within the submucosa, these findings are not specific, and histopathological evaluation remains the gold standard for diagnosis [6, 22]. This case highlights a key diagnostic pitfall: displacement of dysplastic glands into the submucosa may mimic true stromal invasion, leading to potential overstaging as invasive carcinoma. Careful assessment of the muscularis mucosae and of the character of the displaced glands is therefore essential to distinguish pseudoinvasion from true invasion and to avoid unnecessary aggressive treatment [23].

In retrospect, the preoperative imaging in this patient was in most respects typical of a GIST, which explains why that diagnosis was favored: a well‐circumscribed subepithelial mass showing progressive, strong enhancement from 37 HU on unenhanced images to 70 HU in the arterial phase and 108 HU in the venous phase is a hypervascular pattern characteristic of such tumors. Two findings, however, were less typical. First, a well‐defined area within the lesion showed no enhancement in any phase, indicating a cystic component; although cystic degeneration is well recognized in GISTs, it is a feature of larger lesions and is distinctly unusual in a tumor of only 22 mm, and in a small subepithelial lesion, it should prompt consideration of cystic entities including GCP, duplication cyst, and heterotopic pancreas. This nonenhancing area corresponded, in retrospect, to the mucin‐filled cysts identified on gross examination. Second, although the overlying mucosa was smooth and intact, an appearance entirely typical of a mesenchymal subepithelial tumor, the biopsy taken from it showed active *Helicobacter pylori*–associated gastritis. This finding is nonspecific in isolation, but it documents the chronic mucosal injury that underlies GCP, and taken together with the cystic component, it would have justified widening the preoperative differential diagnosis beyond a mesenchymal tumor. Third, endoscopic ultrasonography was not obtained in this case; had it been performed, the demonstration of anechoic multicystic spaces within a thickened second and third layer would have been the single most informative preoperative finding and might have redirected management toward endoscopic submucosal dissection rather than partial gastrectomy [6, 22]. Our experience suggests that a nonenhancing cystic component within a small gastric subepithelial lesion, particularly in a patient with active *Helicobacter pylori* gastritis, should prompt endoscopic ultrasonography and, where feasible, deep or tunnel biopsy before a mesenchymal tumor is assumed.

The preoperative impression of a GIST rested on a limited set of findings: a well‐demarcated subepithelial mass with a smooth, intact overlying mucosa in a patient whose superficial biopsy showed only gastritis, together with progressive hypervascular enhancement on computed tomography. These features are supportive but not specific, and a broader differential should have been entertained. Leiomyoma, which is common at the cardia, typically shows homogeneous and relatively low‐level enhancement and arises from the muscularis mucosae or muscularis propria; schwannoma usually shows homogeneous, progressive enhancement with perilesional lymphoid cuffing; ectopic pancreas favors the antrum, is often ovoid with an indistinct border, and may show a duct‐like opening; and duplication cyst is anechoic with a layered wall and does not enhance internally. GCP differs from all of these in that its cystic spaces are confined to a thickened submucosa, are typically multiple and small rather than unilocular, and are accompanied by an intact overlying muscularis propria; the CT counterpart is a submucosal mass with nonenhancing hypoattenuating foci within an enhancing solid component, as seen here [19]. In our patient, the internal nonenhancing focus was the finding that did not fit a mesenchymal tumor.

The absence of endoscopic ultrasonography also deserves comment. Endoscopic ultrasonography defines the layer of origin, internal echogenicity, cystic content, and vascularity and in GCP characteristically shows anechoic multicystic spaces within a thickened second and third layer [6, 22]. Had it been performed in our patient, this pattern might have been demonstrated, raising suspicion for GCP and narrowing the differential diagnosis away from a mesenchymal tumor; several reported GCP‐associated neoplasms of comparable size have been managed successfully by endoscopic submucosal dissection rather than gastrectomy (Table 1). However, endoscopic ultrasonography features alone cannot definitively exclude a mesenchymal tumor [24], and whether tissue acquisition would still have been required, or whether our own management would ultimately have differed, cannot be established with certainty. We nonetheless acknowledge the absence of endoscopic ultrasonography as a limitation of our management. The decision to proceed directly to surgery followed from the lesion measuring 22 mm, from the patient being symptomatic, and from the multidisciplinary tumor board considering the endoscopic and computed tomographic findings sufficient for an operative indication. Lesions ≥ 2 cm are generally considered candidates for resection given their increased malignant potential; however, this threshold is not absolute, and the decision should be individualized based on endoscopic ultrasonography findings, lesion characteristics, presence of symptoms, and the availability of local endoscopic or surgical expertise [24]. In retrospect, endoscopic ultrasonography should have preceded that decision even when a lesion of this size is destined for resection, because it determines whether resection can be endoscopic rather than surgical.

The nomenclature of this lesion deserves comment. GCP, diffuse cystic malformation, and submucosal heterotopic gastric gland have all been applied to lesions in which gastric glands are found beneath the muscularis mucosae, and the term heterotopic submucosal gastric gland has been used increasingly in the Japanese and international literature, including for dysplastic and carcinomatous lesions arising within such glands [25, 26]. Recent molecular data indicate that many of these lesions are clonal proliferations harboring activating KRAS or GNAS mutations rather than purely reactive changes, and that the small submucosal glandular lesions conventionally termed GCP lie at one end of a single morphological spectrum, with gastric inverted polyps at the other [25]. We have retained the term GCP for the present lesion for three reasons: The displaced glands remained in continuity with the surface epithelium rather than lying isolated within the submucosa; they were enveloped by smooth muscle traceable to the muscularis mucosae, a configuration indicating prolapse of native mucosa rather than a separate submucosal glandular population; and the patient had active *Helicobacter pylori* gastritis, providing an acquired mechanism for glandular displacement. We acknowledge that this distinction is not always reproducible in individual cases and that the two terms are frequently used interchangeably; what is diagnostically critical, irrespective of nomenclature, is the recognition that the submucosal glands in this setting are displaced rather than invasive.

The principal histological differential diagnosis is gastric inverted hyperplastic (hamartomatous inverted) polyp, which likewise shows downward extension of foveolar and pyloric‐type glands accompanied by smooth muscle and frequent cystic dilatation, and which several authors regard as closely related to, or part of the same spectrum as, GCP [13, 27, 28]. Features favoring an inverted hyperplastic polyp include a discrete, largely submucosal nodular growth of well‐organized mucosa inverted through a focal defect in the muscularis mucosae with only a narrow connection to the surface, a predominance of nondysplastic hyperplastic foveolar epithelium with edematous lamina propria, and admixed pyloric glands with, in some cases, parietal and chief cells [27]. In the present case, the lesion did not form such a discrete inverted nodule: The dilated glands extended broadly beneath the mucosa over a wide front and herniated through multiple separate defects in the muscularis mucosae rather than through a single focal defect, remained in continuity with the overlying dysplastic surface epithelium, and comprised a mixture of high‐grade dysplastic and nondysplastic glands on a background of active *Helicobacter pylori* gastritis. These features favored GCP with high‐grade dysplasia over an inverted hyperplastic polyp with secondary dysplastic change.

The pathogenesis of GCP remains controversial. Several studies have reported that GCP may develop many years after abdominal surgery, particularly Billroth II reconstruction [5, 23]. These procedures are thought to weaken the gastric wall through mechanical and inflammatory injury, thereby promoting the prolapse of hyperplastic glands. In addition, bile reflux following Billroth II surgery may contribute to glandular displacement by inducing chronic mucosal irritation at the anastomotic site [23]. Conversely, GCP occurring in patients without prior surgery has been associated with chronic mucosal injury related to peptic ulcer disease and/or *Helicobacter pylori* infection [29, 30]. Epstein–Barr virus infection has also been implicated in some series, although its interaction with *Helicobacter pylori* remains unclear and requires further investigation [31].

In the present patient, these mechanisms can be examined directly. He had no history of gastric surgery but was *Helicobacter pylori*–positive, with histologically confirmed active chronic gastritis, so *Helicobacter pylori*–associated chronic mucosal injury is the most plausible pathogenic mechanism. Repeated cycles of epithelial injury, erosion, and regenerative hyperplasia are thought to disrupt and splay the muscularis mucosae, permitting herniation of regenerating glands into the submucosa, where impaired drainage leads to cystic dilatation and mucin retention. This sequence is consistent with the mucin‐filled cysts identified on gross examination and with the active chronic gastritis documented in the adjacent mucosa. The same chronic inflammatory milieu also provides a plausible route to neoplasia: *Helicobacter pylori*–driven atrophic gastritis and intestinal metaplasia constitute the established precursor pathway to gastric dysplasia, and the p53‐null pattern with a high Ki‐67 index observed here indicates that TP53 inactivation had already occurred within the displaced glands. Whether the dysplasia arose de novo within the submucosal glands or developed in the surface mucosa and extended into preexisting cystic glands cannot be determined with certainty; the continuity between the dysplastic surface epithelium and the submucosal glands in this case favors the latter. Finally, in contrast to series in which GCP‐associated dysplasia and carcinoma have been linked to Epstein–Barr virus and to aberrant p53 expression [31, 32], Epstein–Barr virus in situ hybridization was negative in our patient, arguing against an Epstein–Barr virus‐driven mechanism and leaving *Helicobacter pylori* as the dominant identifiable etiological factor.

Currently, there is no standardized treatment for GCP‐associated neoplasia, as most cases are diagnosed incidentally following surgical or endoscopic resection performed for suspected malignancy. However, available data suggest a relatively favorable prognosis, likely due to early‐stage detection [33]. These observations highlight the importance of accurate preoperative assessment and awareness of this entity to avoid unnecessary aggressive treatment.

Follow‐up in the reported cases ranged from 5 to 120 months, and all patients with available data remained free of disease, but no series has followed a cohort of GCP‐associated neoplasms long enough to quantify the risk of local recurrence, metachronous gastric neoplasia, or subsequent carcinoma. The 13‐month follow‐up available for our patient is correspondingly short, and we acknowledge that no conclusion about long‐term outcome can be drawn from it. Two considerations nevertheless argue for sustained surveillance rather than discharge. First, the underlying field abnormality, *Helicobacter pylori*–associated chronic gastritis with the atrophy and intestinal metaplasia that accompany it, persists after resection of the index lesion and carries a continuing risk of metachronous gastric neoplasia. Second, residual GCP outside the resection margin cannot be excluded on the basis of a single specimen. Our patient has accordingly been kept under endoscopic surveillance with biopsy of the anastomosis and the remnant gastric mucosa every 3 months for the first 6 months and every 6 months thereafter, supplemented by *Helicobacter pylori* eradication with confirmation of cure and by cross‐sectional imaging only when symptoms or endoscopic findings warrant it. At the most recent endoscopy, 13 months after resection, there was no evidence of residual or recurrent dysplasia, of metachronous neoplasia, or of lymphadenopathy. We intend to continue 6‐monthly endoscopy to at least 5 years. Given the rarity of the condition, this schedule is extrapolated from surveillance practice after endoscopic resection of early gastric neoplasia rather than derived from GCP‐specific evidence.

## 4. Conclusion

GCP is a rare gastric lesion that may mimic GISTs on endoscopy and imaging. High‐grade dysplasia arising in GCP is an uncommon but important precursor lesion that often presents as a submucosal mass, making preoperative diagnosis challenging and highlighting the essential role of histopathological confirmation. This case underscores a key diagnostic pitfall, in which glandular displacement into the submucosa may simulate invasive carcinoma and lead to potential overstaging. In our case, the muscularis mucosae was focally discontinuous, so that continuity of the muscle layer alone could not settle the question. What excluded true submucosal invasion was the finding that dysplastic and nondysplastic glands had been displaced together, within smooth muscle derived from the muscularis mucosae and accompanied by lamina propria‐type stroma, in the absence of desmoplasia or infiltrative glandular contours. Recognizing this averted overstaging as invasive adenocarcinoma and the additional oncological treatment that would have followed. Although no standardized treatment has been established, both surgical and endoscopic approaches appear to yield favorable outcomes, likely due to early‐stage detection.

## Author Contributions

Vy Thi‐Thuy Duong contributed to the conception and design of the study, data collection, histopathological evaluation, and manuscript drafting.

Anh The Phan contributed to clinical data collection and interpretation.

Tho Ngoc‐Quynh Le contributed to histopathological evaluation, manuscript revision, and final approval.

Giang Huong Tran contributed to histopathological evaluation, manuscript revision, and final approval.

Huyen Thi Thanh Le contributed to histopathological evaluation, manuscript revision, and final approval.

## Funding

The authors declare that no funding was received for this study.

## Disclosure

All authors read and approved the final manuscript.

## Ethics Statement

The authors have nothing to report.

## Consent

Written informed consent for publication of the relevant information and images was obtained from the patient.

## Conflicts of Interest

The authors declare no conflicts of interest.

## Data Availability

The data that support the findings of this study are available upon request from the corresponding author. The data are not publicly available due to privacy or ethical restrictions.
